# Interferon-Driven Immune Dysregulation in Common Variable Immunodeficiency–Associated Villous Atrophy and Norovirus Infection

**DOI:** 10.1007/s10875-022-01379-2

**Published:** 2022-10-25

**Authors:** Valentina Strohmeier, Geoffroy Andrieux, Susanne Unger, Anna Pascual-Reguant, Adam Klocperk, Maximilian Seidl, Otavio Cabral Marques, Marleen Eckert, Katja Gräwe, Michelle Shabani, Caroline von Spee-Mayer, David Friedmann, Ina Harder, Sylvia Gutenberger, Baerbel Keller, Michele Proietti, Alla Bulashevska, Bodo Grimbacher, Jan Provaznik, Vladimir Benes, Sigune Goldacker, Christoph Schell, Anja E. Hauser, Melanie Boerries, Peter Hasselblatt, Klaus Warnatz

**Affiliations:** 1 Department of Rheumatology and Clinical Immunology, Medical Center - University of Freiburg, Faculty of Medicine, University of Freiburg, Freiburg, Germany; 2 Center for Chronic Immunodeficiency (CCI), Medical Center - University of Freiburg, Faculty of Medicine, University of Freiburg, Freiburg, Germany; 3 Faculty of Biology, University of Freiburg, Freiburg, Germany; 4 Institute of Medical Bioinformatics and Systems Medicine, Medical Center - University of Freiburg, Faculty of Medicine, University of Freiburg, Freiburg, Germany; 5 Department of Rheumatology and Clinical Immunology, Charité - Universitätsmedizin Berlin, corporate member of Freie Universität Berlin and Humboldt-Universität Zu Berlin, Charitéplatz 1, 10117 Berlin, Germany; 6 Immune Dynamics, Deutsches Rheuma-Forschungszentrum (DRFZ), a Leibniz Institute, Charitéplatz 1, 10117 Berlin, Germany; 7 Department of Immunology, 2Nd Faculty of Medicine, Charles University and University Hospital in Motol, Prague, Czech Republic; 8 Institute for Surgical Pathology, University Medical Center Freiburg, Freiburg, Germany; 9 Institute of Pathology, Heinrich Heine University and University Hospital of Dusseldorf, Dusseldorf, Germany; 10 Department of Immunology, Institute of Biomedical Sciences, University of São Paulo, São Paulo, SP Brazil; 11 Department of Clinical and Toxicological Analyses, School of Pharmaceutical Sciences, University of São Paulo, São Paulo, SP Brazil; 12 Network of Immunity in Infection, Malignancy, and Autoimmunity (NIIMA), Universal Scientific Education and Research Network (USERN), São Paulo, SP Brazil; 13 RESIST – Cluster of Excellence 2155 to Hanover Medical School, Satellite Center Freiburg, Freiburg, Germany; 14 DZIF – German Center for Infection Research, Satellite Center Freiburg, Freiburg, Germany; 15 CIBSS – Centre for Integrative Biological Signalling Studies, Albert-Ludwigs University, Freiburg, Germany; 16 European Molecular Biology Laboratory (EMBL), Genomics Core Facility, Heidelberg, Germany; 17 German Cancer Consortium (DKTK) and German Cancer Research Center (DKFZ), Partner Site Freiburg, 79110 Freiburg, Germany; 18 Department of Medicine II, Medical Center - University of Freiburg, Faculty of Medicine, University of Freiburg, Freiburg, Germany; 19 Department of Pharmacy and Postgraduate Program of Health and Science, Federal University of Rio Grande do Norte, Natal, Brazil

**Keywords:** CVID, Villous atrophy, Norovirus, Duodenum, Interferon response genes, Cytotoxic T cell response

## Abstract

**Purpose:**

About 15% of patients with common variable immunodeficiency (CVID) develop a small intestinal enteropathy, which resembles celiac disease with regard to histopathology but evolves from a distinct, poorly defined pathogenesis that has been linked in some cases to chronic norovirus (NV) infection. Interferon-driven inflammation is a prominent feature of CVID enteropathy, but it remains unknown how NV infection may contribute.

**Methods:**

Duodenal biopsies of CVID patients, stratified according to the presence of villous atrophy (VA), IgA plasma cells (PCs), and chronic NV infection, were investigated by flow cytometry, multi-epitope-ligand cartography, bulk RNA-sequencing, and RT-qPCR of genes of interest.

**Results:**

VA development was connected to the lack of intestinal (IgA^+^) PC, a T helper 1/T helper 17 cell imbalance, and increased recruitment of granzyme^+^CD8^+^ T cells and pro-inflammatory macrophages to the affected site. A mixed interferon type I/III and II signature occurred already in the absence of histopathological changes and increased with the severity of the disease and in the absence of (IgA^+^) PCs. Chronic NV infection exacerbated this signature when compared to stage-matched NV-negative samples.

**Conclusions:**

Our study suggests that increased IFN signaling and T-cell cytotoxicity are present already in mild and are aggravated in severe stages (VA) of CVID enteropathy. NV infection preempts local high IFN-driven inflammation, usually only seen in VA, at milder disease stages. Thus, revealing the impact of different drivers of the pathological mixed IFN type I/III and II signature may allow for more targeted treatment strategies in CVID enteropathy and supports the goal of viral elimination.

**Graphical abstract:**

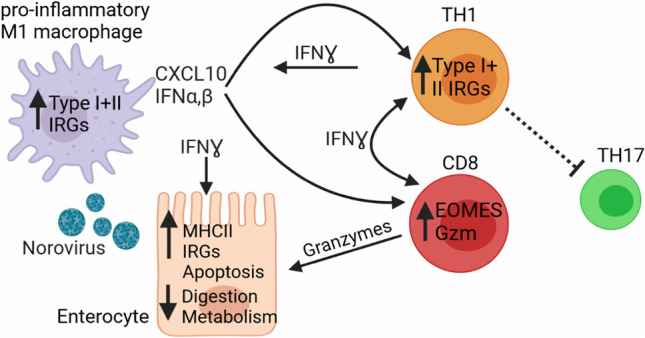

**Supplementary Information:**

The online version contains supplementary material available at 10.1007/s10875-022-01379-2.

## Introduction

Common variable immunodeficiency (CVID) is a rare, inborn, heterogeneous antibody deficiency syndrome with a prevalence of about 1:25,000 [1]. In about 30% of CVID patients, the immunodeficiency not only disrupts the humoral response against pathogens, but is also associated with autoimmunity and inflammatory organ disease [2, 3]. Beside an increased risk of gastrointestinal infection by salmonella, campylobacter, giardia, or norovirus (NV), non-infectious chronic gastrointestinal (GI) disease occurs in 9–24% of patients, rendering the frequency in CVID at least 10 times higher than the prevalence for inflammatory bowel disease (IBD) and celiac disease (CD) in the normal German population [3–7]. Diarrhea and malabsorption are the most common symptoms [8], contributing to higher morbidity and mortality observed in CVID patients with GI disease [2]. The standard treatment of CVID through immunoglobulin replacement therapy has little effect on the non-infectious gut disease [8], and therefore, many patients receive topical steroids or non-specific immunosuppressive treatment for this complication, potentially aggravating the already impaired local and systemic host defense. The term “CVID enteropathy” has been frequently, yet inconsistently used for patients with chronic GI disease, ranging from chronic persistent diarrhea to CD-like autoimmune enteropathy [5].

The disease can affect both the upper and the lower GI tract. Frequent histopathologic findings in intestinal biopsies include increased epithelial apoptosis, increased intraepithelial lymphocytes (IELs) and villous atrophy (VA) of the upper gastrointestinal tract mimicking CD or autoimmune enteropathy, lymphoid hyperplasia affecting the small and the large intestine, and microscopic colitis or crypt distortion and granuloma in a few patients, resembling Crohn’s-like disease of the colon [8–12]. In contrast to CD, VA, which is reported in 30–51% of CVID patients with GI complaints, is rarely associated with HLA-DQ2 or HLA-DQ8 and does not respond to gluten-free diet [13].

Immunologically, CVID patients typically present with reduced numbers of plasma cells (PCs), especially IgA PCs, in the intestine [8, 10, 14]. Beside the potential contribution of absent mucosal IgA to the disturbed GI barrier function, a small British study had suggested that chronic NV infection might be linked to VA development [15]. Recent studies of CVID enteropathy have suggested that alterations of the intestinal microbiota are associated with an increased interferon (IFN)-driven inflammation and altered lipid metabolism in the GI mucosa [16, 17]. The source of the increased IFN signature, its association with the progression of the disease, and the contribution of the lack of IgA remain unclear. Also, the impact of chronic NV infection on the altered transcriptome in CVID enteropathy has not been investigated so far.

We therefore performed a cross-sectional study with 65 CVID enteropathy patients, of which 21 were affected by VA, to dissect the local small intestinal immune dysregulation at different stages of CVID-associated celiac-like enteropathy. We conducted flow cytometric, transcriptomic, and multiplex immunofluorescence analysis of the affected tissues, focusing on the effects of the presence versus the absence of VA, chronic NV infection, and plasma cells, aiming to explore potentially useful signatures for diagnosis and a better understanding of the pathogenesis of this deleterious disease manifestation.

## Methods

### Patients and Controls

Seventy-two duodenal biopsies were obtained from 65 CVID patients with GI symptoms (diarrhea, defined as more than three loose stools/day for more than 3 days at a time; weight loss; or abdominal pain) undergoing diagnostic upper endoscopy between March 2014 and May 2022 at the Freiburg University Medical Center. Seven patients were included twice because they changed their VA/noVA status during the observation period (see Table S1). In the majority of patients, stool samples were screened shortly before or at the time point of endoscopic procedure for the presence of norovirus (genotypes I and II), rotavirus, astrovirus, and adenovirus by multiplex PCR. Chronic infection with NV was diagnosed, if patients had previously been tested repeatedly positive for NV, over a time period of at least 10 months. All patients fulfilled the criteria for CVID according to the European Society for Immunodeficiencies (ESID) (www.esid.org). CVID-associated monogenic defects were diagnosed in 17 patients identifying mutations in *CTLA4* (*n* = 5), *ICOS* (*n* = 1), *NFKB1* (*n* = 5), *NFKB2* (*n* = 1), *LRBA* (*n* = 1), *PI3K* (*n* = 1), *TNFRSF13B* (TACI, *n* = 1), *TNFRSF13C* (BAFF receptor, *n* = 1), and *TNFRSF6* (FAS, *n* = 1). The following extended clinical data was recorded for all CVID patients: diarrhea, splenomegaly, generalized lymphadenopathy, granulomatous disease, autoimmune cytopenias, interstitial lung disease, liver disease, and use of systemic and local steroids. All patients were of Caucasian ethnic background. Fifty percent of the patients were female. Mean age at biopsy was 43 years. There was no significant difference in age or gender distribution between VA and noVA patients, as the VA group presented with 43% females and a mean age of 43 years and the noVA group with 53% females and a mean age of 42.5 years. All patients were under regular immunoglobulin replacement therapy. Ten patients had been treated with rituximab in the past, but not within the last 12 months before the endoscopy (detailed information on single patients is listed in Table S1). Patients who were under immunosuppressive therapy during the time point of endoscopic procedure or that suffered from active CMV infection (defined by blood and histological analysis) were excluded from this study. Control duodenal biopsies were obtained from 23 individuals without known immunodeficiency or inflammatory small intestinal disease, undergoing upper endoscopy for diagnostic purposes (for further information on diagnostic question and final diagnosis see Table S2). Seventy percent of healthy controls (HCs) were female, and mean age at endoscopic procedure was 49 years (for further information on HCs, see Table S1). The study was approved by local authorities (Freiburg 526/14 and amendment 26/14_170104). All procedures performed in this study were in accordance with the ethical standards of the institutional and national research committee and with the 1964 Helsinki Declaration and its later amendments, and written informed consent was obtained from all patients and controls.

### Histological Classifications

For histopathological analysis, 3-µm sections were taken from buffered formalin-fixed paraffin-embedded (FFPE) biopsies and stained with hematoxylin and eosin (H&E). Photos were taken with an Olympus BX51 microscope (Olympus) with the AxioCam MRc microscope camera (Carl Zeiss). The following histological criteria were graded either present or absent in 30 patient-derived tissues: chronic crypt architectural distortion if ≥ 2 or more crypts were affected, crypt hyperplasia, acute cryptitis (≥ 2 granulocytes per at least on crypt diameter), crypt abscesses, erosions, eosinophilia (> 10 eosinophils per 400 × field of view), intraepithelial lymphocytosis (≥ 25 lymphocytes per 100 epithelial cells, according to Hayat et al. [18]), lymphoid hyperplasia (defined as present follicular structures with or without germinal centers), and granulomas (defined as an epithelioid and/or giant cell aggregate).

All 72 duodenal biopsies were classified according to the Marsh [19] Oberhuber score [20], without (Marsh 0) or with increased intraepithelial lymphocytes (Marsh 1 and 2) being scored as ≥ 30 lymphocytes per 100 epithelial cells. Villous atrophy was graded as slight (< 30% of villi, Marsh 3-a), medium (30–80% of villi, Marsh 3-b), or severe (> 80% of villi, Marsh 3-c). Patients with Marsh 0 to 2 were classified as CVID noVA, and patients with Marsh 3-a to Marsh 3-c were classified as CVID VA. The presence or absence of total plasma cells (for 63 samples), as well as of IgA, IgG, and IgM plasma cells (for 61 samples), was graded either present or absent within duodenal tissues (further information on single patients is depicted in Table S1).

### Immunohistochemistry

Immunohistochemistry was carried out on 2-µm sections from formalin-fixed paraffin-embedded biopsies. Staining for IFN-γ was performed after heat-mediated epitope retrieval in a pressure cooker for 2 min in citric buffer (pH 6) and after blocking for 5 min with H_2_O_2_ (included in EnVision Flex, Dako). Anti-IFN-γ antibody (clone E-10, Santa Cruz Biotechnology) was used at a 1:50 dilution and visualized using a horseradish peroxidase–catalyzed, DAB-based brown chromogen reaction kit (EnVision Flex Kit, Dako). Tissue was evaluated for positive cells in a semi-quantitative way: 0 = none, 1 = 1–10%, 2 = 11–50%, 3 = 51–80%, and 4 = 81–100% of cells. Staining for IgA/IgG/IgM was performed after heat-mediated epitope retrieval in a steamer for 20 min in citric buffer (pH 9; Dako) and after blocking for 10 min with H_2_O_2_ (included in EnVision Flex Kit, Dako) with ready-to-use polyclonal rabbit (Rb) anti-human IgA (IR510, Dako), IgG (A0423, Dako), or IgM (IR513, Dako) antibodies. Antibody binding was visualized in the same way by the EnVision Flex Kit (Dako). IgA, IgG, and IgM plasma cells were graded either present or absent in the tissue sections.

### Processing of Tissue

T cell isolation from intestinal biopsies was performed by mechanical disruption. The tissue was mashed through a 40-µm mesh, and cells were resuspended in RPMI medium with 10% FCS (Merck). Macrophage isolation was performed by epithelial cell removal with PBS supplemented with 5% FCS, 10 mM HEPES, 10 U/mL penicillin, 100 µg/mL streptomycin (all Gibco), and 2 mM EDTA (Invitrogen) for 15 min at 37°, followed by enzymatic isolation with 1 mg/mL r 1 h at 37°.

### FACS

Flow cytometric surface staining occurred for 15 min at 4° in the dark. For macrophages, Fc receptor blocking (TruStain FcX BioLegend) was performed for 10 min at RT, prior to surface staining.

For intracellular cytokine measurements, isolated intestinal cells were stimulated with 5 ng/mL PMA and 750 ng/mL ionomycin in the presence of brefeldin A (all Sigma-Aldrich) for 4 h, at 37 °C. For intracellular staining, the Cytofix/Cytoperm™ Fixation/Permeabilization Solution Kit (BD Biosciences) was used according to the manufacturer’s instructions. For intracellular staining of granzyme B, isolated intestinal cells were stained for surface receptors and live/dead (Zombie UV, BioLegend) dye, followed by fixation and permeabilization with the eBioscience™ Foxp3/Transcription Factor Staining Buffer Set (Thermo Fisher) and intracellular staining for granzyme B, according to the manufacturer’s instructions. Data were acquired on a Gallios® flow cytometer (Beckman Coulter) or LSRFortessa™ (Becton Dickinson) and analyzed using FlowJo® software (version 7.6.5 or 10). Antibodies used for flow cytometry are listed in Table S3.

### Multi-Epitope-Ligand Cartography

#### Tissue Preparation for Multi-Epitope-Ligand Cartography

Fresh frozen tissue was cut in 5-µm sections with a MH560 cryotome (Thermo Fisher, Waltham, MA, USA) on 3-aminopropyltriethoxysilane (APES)-coated cover slides (24 × 60 mm; Menzel-Gläser, Braunschweig, Germany). Samples were fixed for 10 min at room temperature (RT) with freshly opened, electron microscopy grade 2% paraformaldehyde (methanol and RNase free; Electron Microscopy Sciences, Hatfield, Philadelphia, PA, USA). After washing, samples were permeabilized with 0.2% Triton X-100 in PBS for 10 min at RT and unspecific binding was blocked with 10% goat serum and 1% BSA in PBS for at least 20 min. Afterwards, a fluid chamber holding 100 µL of PBS was created using “press-to-seal” silicone sheets (1.0 mm thickness; Life Technologies, Carlsbad, CA, USA) with a circular cut-out (10 mm diameter), which was attached to the cover slip surrounding the sample. Prior to each multi-epitope-ligand cartography (MELC) experiment, fresh washing solution consisting of PBS with 2% BSA and 0.02% Triton X-100 was prepared. The sample was placed on the sample holder and fixed with adhesive tape followed by accurate positioning of the binning lens, the light path, as well as Köhler illumination of the microscope.

#### MELC Image Acquisition

MELC image acquisition was performed as previously shown [21, 22]. In short, multiplexed histology data was generated on a modified Toponome Image Cycler® MM3 (TIC) originally produced by MelTec GmbH & Co. KG [23]. The ImageCycler is a robotic microscopic system that consists of (I) an inverted widefield (epi) fluorescence microscope Leica DM IRE2 equipped with a CMOS camera and a motor-controlled XY stage, (II) Cavro XL3000 Pipette/Diluter (Tecan GmbH), and (III) a software program (MelTec TIC-Control) for controlling of the microscope and pipetting system and for synchronized image acquisition.

A MELC experiment is a sequence of iterative cycles, each consisting of four steps: (i) pipetting of the fluorescence-coupled antibody onto the sample, incubation, and subsequent washing; (ii) cross-correlation autofocusing based on phase-contrast images, followed by acquisition of the fluorescence images as 3D stacks (± 5 z-steps); (iii) photo-bleaching of the fluorophore; and (iv) a second autofocusing step followed by acquisition of a post-bleaching fluorescence image 3D stack (± 5 z-steps). In each four-step cycle, up to three fluorescence-labeled antibodies were used, combining PE, FITC, and DAPI. The antibodies used for MELC staining are listed in Table S4.

#### MELC Image Pre-processing

Image pre-processing was conducted as previously described [22]. In short, the reference phase-contrast image taken at the beginning of the measurement was used to align all images by cross-correlation. Afterwards, the signal of the bleaching image in each cycle and focal plane was used to subtract the background and correct the illumination of the fluorescence image obtained in the same cycle and focal plane [23]. Subsequently, an “Extended Depth of Field” algorithm was applied on the 3D fluorescence stack in each cycle [24]. Images were then normalized in Fiji [25], where a rolling ball algorithm was used for background estimation; edges were removed (accounting for the maximum allowed shift during the autofocus procedure); and fluorescence intensities were stretched to the full intensity range (16 bit =  > 216).

#### Cell Segmentation and Cell Type Identification

Segmentation was performed in a two-step process, a signal classification step using Ilastik 1.3.2 [26] followed by an object recognition step using CellProfiler 4.2.1 [27], as previously described [22]. Ilastik was used to classify pixels into three classes (nuclei, membrane, and extracellular matrix (ECM)) and to generate probability maps. A combination of images (CD45, pancytokeratin and HLA-DR, HLA-DP, HLA-DQ) was summed up and used to classify cell membranes, while only the DAPI image was used to classify nuclei. All pixels not belonging to membranes or nuclei were classified as ECM. The random forest algorithm (machine-learning, Ilastik) was trained by manual pixel classification in a small region of a dataset (approx. 6% of the image). The rest of the dataset as well as fifteen extra datasets analyzed here were classified without re-training, using the random forest algorithm. The nuclei, the membrane, and the ECM probability maps obtained were subsequently used in CellProfiler to segment the nuclei and membranes and to generate nuclei and cellular binary masks, respectively. These masks were superimposed on the individual fluorescence images acquired for each marker included in the MELC run, in order to extract single-cell information for individual markers, i.e., median fluorescence intensity (MFI) of each marker per segmented cell. Based on the extracted single-cell MFI values and by visual inspection and thresholding of the images, cells were computationally classified into relevant cell types and quantified. Those cells with CD3 MFI > 0.02 and CD8 MFI > 0.02 were classified as CD8^+^ T cells, those with CD3 MFI > 0.02 and CD4 MFI > 0.1 were classified as CD4^+^ T cells, those with CD3 MFI > 0.02 and CD8 MFI > 0.02 or CD4 MFI > 0.1 were classified as total T cells, and those with pancytokeratin MFI > 0.02 were classified as epithelial cells. For both CD8^+^ and CD4^+^ T cell subsets, we also classified and quantified the phosphorylated STAT1 (pSTAT1)^+^ cells by setting a threshold at 0.02. Granzyme B (GzmB)^+^CD8^+^ T cells were classified with CD3 MFI > 0.02, CD8 MFI > 0.2, and GzmB MFI > 0, and GzmB^+^CD103^+^CD8^+^ T cells were classified with CD3 MFI > 0.02, CD8 MFI > 0.2, GzmB MFI > 0, and CD103 MFI > 0.

### Reverse Transcription Quantitative Polymerase Chain Reaction

Total RNA from tissues was extracted using the RNeasy Mini Kit (Qiagen) according to the manufacturer’s instructions. Complementary DNA (cDNA) was generated using 300 ng RNA, Superscript III, and random hexamer primers (both Invitrogen) following standard protocols. Quantitative RT-PCR was performed using SYBR Green Dye (Roche) on the Roche LightCycler 480. Results were normalized to the tissue housekeeping gene ubiquitin + tubulin. Relative expression levels were calculated as 2^(Ct (mean of HK1 and HK2)^ ^−^ ^Ct (gene))^. Primers were designed using Primer3Plus [28] (Table S5) and ordered from eurofinsgenomics.eu.

### RNA-Sequencing

Duodenal biopsies were stored in RNAlater (Qiagen) and subsequently dispersed using a homogenizer (T10, IKA). RNA was extracted using RNeasy Plus Mini Kit (Qiagen) according to the manufacturer’s instructions. RNA integrity and concentration was assessed using the Agilent 2100 Bioanalyzer RNA Nano Chip, and only samples with a RNA integrity number (RIN) above 8 were proceeded. Barcoded stranded mRNA-seq libraries were prepared from high-quality total RNA samples (~ 200 ng/sample) using the TruSeq RNA Sample Preparation v2 Kit (Illumina) implemented on the liquid handling robot Beckman FXP2. Obtained libraries that passed the QC step were pooled in equimolar amounts; 1.8 pM solution of this pool was loaded on the Illumina sequencer NextSeq 500 and sequenced unidirectionally, generating ~ 500 million reads, each 85 bases long.

### RNA-Sequencing Data Analysis

Reads were first trimmed, using Trimmomatic [29], to remove adapter content and bad quality reads. STAR [30] aligner was used to align reads to the human genome (hg19) and quantify read count per gene. Differential gene expression analysis was performed with the DESEq2 R package [31], using the Wald test. Adjusted *P* value (Benjamini-Hochberg) below 0.05 was considered as significance threshold. Gene set enrichment of regulated genes was done with Fisher’s exact test, where gene sets were taken from MSigDB database [32]. Adjusted *P* value below 0.05 was considered as significance threshold. Cell type deconvolution was performed with immunedeconv R package [33] using the Cibersort method [34].

### Statistical Information

Statistical analyses were performed using GraphPad 9 software (GraphPad Software, Inc.). Datasets were tested for normal distribution, and statistical comparisons were done using an unpaired *t* test or the Mann–Whitney test. For comparison of multiple groups, the Kruskal–Wallis or ordinary one-way ANOVA test was used based on normality of datasets. Tukey’s multiple comparison test or Dunn’s correction for multiple testing was used. *P* values of less than 0.05 were considered significant (*P < 0.05, **P < 0.01, ***P < 0.001, ****P < 0.0001). Error bars in all figures define the mean ± standard deviation.

## Results

### T Helper Cell Imbalance and Increased IFN-γ Production in CVID Enteropathy Tissues with Villous Atrophy

**Fig. 1:**
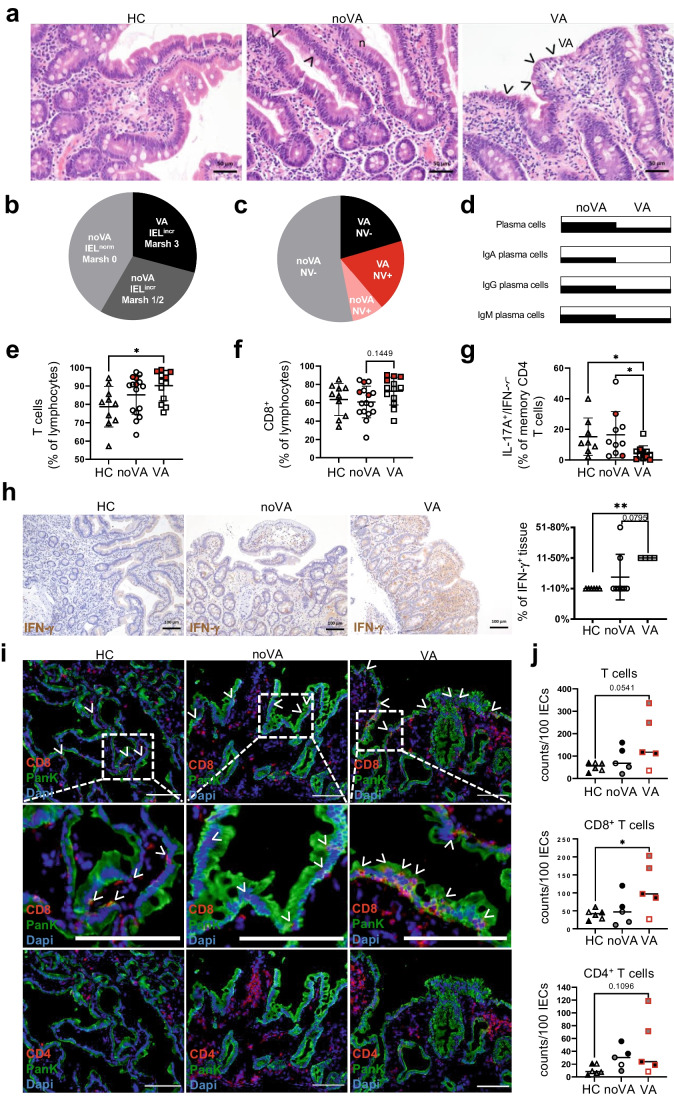
CVID villous atrophy tissues lack (IgA^+^) plasma cells, present with a disturbed T cell homeostasis and increased IFN-γ expression. **a** Representative histologies of duodenal biopsies of a healthy control (HC), a CVID patient without villous atrophy (noVA), and a CVID patient with villous atrophy (VA). *Arrows* denote intraepithelial lymphocytes (IELs). **b** Frequency of patients with increased IELs and VA, according to Marsh 3 (*n* = 21), of patients with increased IELs but absence of VA (noVA), according to Marsh 1–2 (*n* = 21), and patients without increased IELs and noVA, according to Marsh 0 (*n* = 30), within the studied cohort. **c** Frequency of norovirus (NV) infection among VA and noVA patients within the studied cohort. In *pink*, NV-positive noVA patients (*n* = 4); in *red*, NV-positive VA patients (*n* = 9); in *gray*, NV-negative noVA patients (*n* = 26); and in *black*, NV-negative VA patients (*n* = 10). **d** Frequency of the presence of total, IgA^+^, IgG^+^, and IgM^+^ plasma cells (PC) within tissues of VA (*n* = 21) and noVA (*n* = 43) patients, investigated by immunohistochemistry (IHC). **e** Proportion of CD3^+^ T cells of CD45^+^ lymphocytes within HC (*n* = 10), noVA (*n* = 16), and VA (*n* = 13) tissues. Patients with NV infection are highlighted in *red*. **f** Proportion of CD8^+^ T cells of CD45^+^ lymphocytes within HC (*n* = 10), noVA (*n* = 16), and VA (*n* = 13) tissues. Patients with NV infection are highlighted in *red*. **g** Proportion of IL17A^+^/IFN-γ^−^ cells of memory CD4^+^ T cells, after the 4-h PMA + ionomycin stimulation, isolated from tissues of HCs (*n* = 8), noVA (*n* = 10), and VA (*n* = 11) patients. Patients with NV infection are highlighted in *red*. **h** Exemplary IHC staining for IFN-γ expression within tissues of a HC, a noVA patient, and a VA patient. Graph showing statistical analysis of the proportion of IFN-γ^+^ cells among the total tissue of HCs (*n* = 6) and noVA (*n* = 8) and VA (*n* = 4) patients. **i** Representative images of immunofluorescence (IF) staining performed by multi-epitope-ligand cartography (MELC) for CD8 (in *red*), CD4 (in *red*, *bottom*), pancytokeratin (Pank, in *green*), and 4′,6-diamidino-2-phenylindol (Dapi, in *blue*) within HC (*n* = 3), noVA (*n* = 3), and VA (*n* = 3) tissues. *Arrowheads* indicate CD8^+^ IELs. *Dotted line* marks selected field for higher magnification shown in *second row*. *Scale bars* show 100 µm. **j** Relative counts of total, CD4^+^, and CD8^+^ T cells per 100 intestinal epithelial cells (IECs) within HC (*n* = 3), noVA (*n* = 3), and VA (*n* = 3) tissues as quantified from MELC data (see the “Methods”). Two fields of view (FOVs) are depicted for each tissue specimen (except two patients). Color code indicates data of FOVs from the same tissue specimen within the group. In *red*, norovirus positive patients. *P* values as determined by one-way ANOVA with Tukey’s multiple comparison test (**e**, **f**, **j**) or Kruskal–Wallis test with Dunn’s multiple comparison test (**g**, **h**), depending if the samples were normally distributed or not, comparing the mean of each column with the mean of every other column. The linear relationship between Marsh score and single measurements was determined by simple linear regression, defining the coefficient of determination (*R*.^2^) (**e**–**g**)

In order to identify distinctive features of enteropathy, we stratified our cohort into CVID patients with VA, defined by a Marsh-Oberhuber score of 3-a to 3-c, and CVID patients without VA (noVA) including Marsh scores of 0 to 2 (Fig. 1a). Small bowel biopsies revealed increased intraepithelial lymphocyte numbers (≥ 30 IELs/100 enterocytes) in 42/72 CVID samples (58.3%, *dark gray and black portion*) and VA in 21/72 CVID samples (29.2%, *black portion*) (Fig. 1b). NV infection was diagnosed in 9/19 VA patients (47.4%, *red portion*) and 4/30 noVA patients (13.3%, *pink portion*) (Fig. 1c). VA and noVA patients showed an equal percentage of *Helicobacter pylori* (6% vs 8%) and other gastrointestinal infections (25% vs 27.5%). VA patients, compared to noVA patients, presented with an elevated prevalence of other CVID-associated clinical complications like autoimmune cytopenia (57% vs 29%), splenomegaly (91% vs 71%), lymphadenopathy (86% vs 59%), interstitial lung disease (62% vs 42%), granuloma formation (38% vs 26%), and liver disease (52% vs 26%). The prevalence of diarrhea as prominent enteropathy symptom was higher in the VA compared to the noVA group (91% vs 71%), as well as concomitant therapy with low-dose systemic (38% vs 20%) or topical (48% vs 33%) steroids. Interestingly, VA patients had a more frequent history of rituximab treatment (24% vs 10%), which might result from the increased incidence of autoimmune cytopenias within this group (Fig. S1a). With regard to histological manifestations, VA tissues contained more frequently crypt distortion and hyperplasia (Fig. S1b) and were lacking more frequently PCs (71% vs 38%) and IgA^+^ PCs (100% vs 67%) than noVA tissues (Fig. 1d).

Flow cytometric analysis revealed a significant increase of CD3^+^ lymphocytic infiltration in duodenal tissues of VA patients, compared to HC tissues (Fig. 1e). These consisted mostly of CD8^+^ T cells (Fig. 1f, gating shown in Fig. S1c). Of note, tissues of patients with ongoing NV infection showed among the highest percentages of total and CD8^+^ T cell infiltration, within their respective noVA/VA group, compatible with an induced recruitment of these cells to the GI tissues upon NV infection. With regard to cytokine production, there was a significant decrease in IL-17A and IL-17A/IFN-γ (double-) producing CD4^+^ memory T helper (TH) cells in VA tissues, while the proportion of IFN-γ-producing CD4^+^ memory subsets was not altered (Fig. 1g and Fig. S1d, gating shown in Fig. S1e). Additionally, when TH subsets were defined by their ex vivo expression of CXCR3 and CCR6, the proportion of TH17 cells was similarly reduced within VA tissues, while TH1, TH2, and TH1/17 subsets were unchanged (Fig. S1f, gating shown in Fig. S1g). However, immunohistochemistry (IHC) demonstrated a significantly increased IFN-γ production in tissues of patients with VA, compared to controls and the majority of noVA patients (Fig. 1h). Beside an overall higher percentage of TH1 cells, no additional changes in the mucosal TH subset composition were observed in NV^+^ tissues (Fig. S1f).

**Fig. 2:**
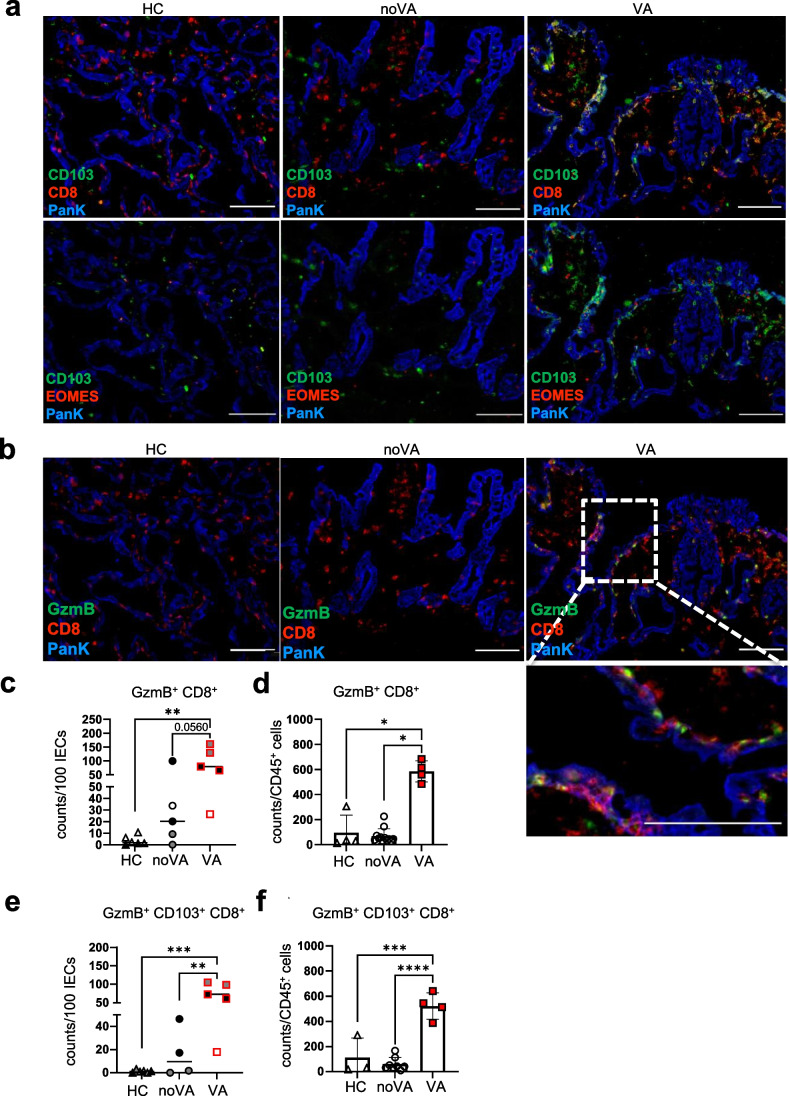
Cytotoxic tissue-resident CD8^+^ T cells accumulate within tissues of CVID VA patients. **a** Representative images of IF staining performed by MELC for CD8 (in *red*, *first row*) or EOMES (in *red*, *second row*), PanK (in *blue*), and CD103 (in *green*). *Scale bars* show 100 µm. **b** Representative images of IF staining performed by MELC for CD8 (in *red*), PanK (in *blue*), and granzyme B (GzmB, in *green*). *Dotted line* marks selected field for higher magnification. *Scale bars* show 100 µm. **c** Relative counts of GzmB^+^CD8^+^ T cells per 100 intestinal epithelial cells (IECs) within HC (*n* = 3), noVA (*n* = 3), and VA (*n* = 3) tissues, analyzed by cell segmentation and cell type identification of MELC. In *red*, NV-positive patients. **d** Relative counts of GzmB^+^CD8^+^ T cells per 1000 CD45^+^ lymphocytes within HC (*n* = 4), noVA (*n* = 12), and VA (*n* = 4) tissues, analyzed by intracellular flow cytometry staining. In *red*, NV-positive patients. **e** Relative counts of GzmB^+^CD103^+^CD8^+^ T cells per 100 IECs within HC (*n* = 3), noVA (*n* = 3), and VA (*n* = 3) tissues, analyzed by cell segmentation and cell type identification of MELC. In *red*, NV-positive patients. **f** Relative counts of GzmB^+^CD103^+^CD8^+^ intraepithelial T cells per 1000 CD45^+^ lymphocytes within HC (*n* = 3), noVA (*n* = 8), and VA (*n* = 4) tissues, analyzed by intracellular flow cytometry staining. In *red*, NV-positive tissues. **c**, **e** Two fields of view (FOVs) are depicted for each tissue specimen (except two tissues). Color code indicates data of FOVs from the same tissue specimen within each group. *P* values as determined by one-way ANOVA with Tukey’s multiple comparison test (**d**–**f**) or Kruskal–Wallis test with Dunn’s multiple comparison test (**c**), depending if the samples were normally distributed or not, comparing the mean of each column with the mean of every other column

Multi-parameter immunofluorescence investigation, by MELC, visualized a distinct spatial distribution of CD4^+^ and CD8^+^ T cells in the duodenal tissues of HCs, CVID noVA patients, and CVID VA patients (Fig. 1i). Within VA tissue, not only more CD8^+^ T cells infiltrated the lamina propria (Fig. 1i, *upper row* and Fig. 1j) but they also localized throughout the epithelial layer and towards the luminal side, whereas in noVA and HC, these cells were mostly mapping the basal border of the epithelial layer (Fig. 1i, *middle row*). CD4^+^ T cells mainly located throughout the subepithelial layer and were expanded only in single patient-derived tissues compared to HC-derived tissues (Fig. 1i, *lower row* and Fig. 1j). Furthermore, in VA tissues, CD8^+^ T cells expressed the IEL marker and integrin CD103 [35], partly together with the transcription factor Eomes (Fig. 2a). Some of the intraepithelial CD8^+^ T cells in the VA tissue appeared positive for the effector molecule GzmB [36] (Fig. 2b). Therefore, GzmB expression within the CD8^+^ T cell compartment was further investigated by MELC and intracellular flow cytometry. In comparison to HCs and nearly noVA tissues, VA tissues presented with a significant expansion of GzmB^+^CD8^+^ T cells (Fig. 2c (MELC) and Fig. 2d (flow cytometry)) especially when gating more specifically on the intraepithelial CD103^+^CD8^+^ T cell subset (Fig. 2e, f). Of note, VA tissues were all derived from patients with chronic NV infection, likely inducing the recruitment of cytotoxic CD8^+^ T cells. Additionally, *GZMB* transcript expression was investigated by reverse transcription quantitative polymerase chain reaction (RT-qPCR) and showed high *GZMB* levels not only in CVID VA, but also in CVID noVA tissues, compared to HCs (Fig. S2a). At the transcriptional level, *GZMB* expression was even already induced in samples with a Marsh-Oberhuber score of 0, when compared to HCs (Fig. S2b). Similar to protein expression, especially NV^+^ tissues showed a highly induced expression of *GzmB* within both enteropathy groups, possibly resulting from the increased activation of NV-specific T_RM_ cells [37] in the infected tissues.

### Activation of Immune and Inflammatory Responses Concurred with Impairment of Intestinal Epithelial Functions in CVID Enteropathy Tissues

**Fig. 3:**
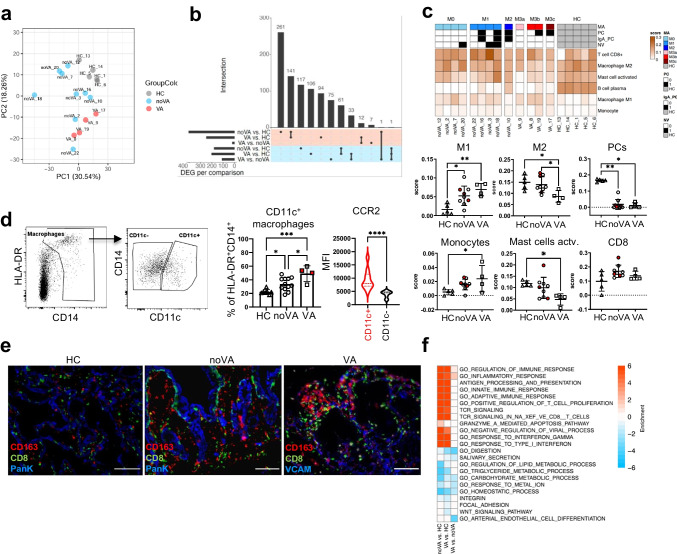
Transcriptome analysis of CVID enteropathy patients reveals immune activation and IFN-driven inflammation in VA and noVA tissues. **a** Principal component analysis (PCA) on RNA-seq data. PCA was performed on the top 10% most variable genes, based on the median absolute deviation (MAD) score calculated on the mRNA-normalized intensity (CPM). Color code indicates the different groups. **b** UpSet plot comparing the differentially expressed genes (adjusted *P* value < 0.05) in noVA vs. HC, VA vs. HC, and VA vs. noVA. Upregulated genes are highlighted with *red* and downregulated genes with *blue background*, respectively. **c** Cell type deconvolution heatmap showing the predicted immune cell distribution in each sample. Color code represents the cell fraction score. Legend depicts information on the Marsh score (M, M0–M3c), presence of total plasma cells (PC) or IgA^+^ PCs (IgA_PC), and presence of norovirus (NV) infection for each patient sample. Statistical analysis for cell fraction scores of M1 and M2 macrophages, PCs, monocytes, activated mast cells, and CD8^+^ T cells (CD8^+^) within HC (*n* = 5), noVA (*n* = 9), and VA (*n* = 4) tissues. Patients with NV infection are highlighted in *red*. **d** Gating strategy for tissue-homing CD11c^+^ and CD11c^−^ macrophage subsets. Pre-gated on living CD45^+^ cells. Statistical analysis of the proportion of CD11c^+^ macrophages of the total HLA-DR^+^CD14^+^ macrophage subset, within HCs (*n* = 7), noVA (*n* = 12), and VA (*n* = 4) tissues. Patients with NV infection are highlighted in *red*. Mean fluorescence intensity (MFI) of CCR2 in CD11c^+^ (in *red*) and CD11c^−^ (in *black*) macrophages, columns include patient (*n* = 8) and HC (*n* = 2) values. **e** IF staining performed by MELC for CD163 (macrophages, in *red*), CD8 (in *green*), and PanK/VCAM (in *blue*) in HC (*n* = 1), noVA (*n* = 1), and VA (*n* = 1) tissues. *Scale bars* show 100 µm. **f** Gene set enrichment heatmap. Relevant gene sets were selected among the top significant ones. Color code represent the enrichment score (i.e., − log10 *P* value for upregulated gene set, + log10 *P* value for downregulated gene sets). *P* values as determined by one-way ANOVA with Tukey’s multiple comparison test (**c** M1, M2, monocytes; **d** CD11c.^+^ macrophages) or Kruskal–Wallis test with Dunn’s multiple comparison test (**c** PCs, mast cells, CD8) depending if the samples were normally distributed or not, comparing the mean of each column with the mean of every other column and by Mann–Whitney test (**d** CCR2 mean)

To further explore underlying mechanisms of VA development in our cohort, we performed transcriptional profiling by RNA-seq, using bulk tissue of duodenal biopsies of patients with VA (*n* = 6), noVA patients (*n* = 9), and HCs (*n* = 6). After exclusion of outliers (defined in Fig. S3a), samples clustered into the three different groups within the principal component analysis (PCA) (Fig. 3a). While there were over 400 differentially expressed genes (DEGs) between each CVID group and HCs, including more than 200 overlapping genes in VA and noVA, the difference between the two CVID groups was more limited, including 19 upregulated and 109 downregulated DEGs in VA, when compared to noVA tissues (Fig. 3b). By applying Cibersort [34], we identified alterations of cell populations according to their specific gene signatures (defining genes are listed in Fig. S3b) in VA tissues compared to noVA and HC tissues (Fig. 3c). Thus, the expression profile of pro-inflammatory M1 macrophages was significantly enriched in VA tissues, compared to HCs, whereas noVA tissues presented with an intermediate abundance. Simultaneously, the M2 macrophage–defining signature was reduced in VA compared to HC tissues. Consistent with our histological observations (Fig. 1d), PC-related signatures were severely diminished in both CVID groups (Fig. 3c). NV infection did not change this cellular expression distribution. Interestingly, flow cytometric investigation on tissue-homing macrophage subsets revealed a significant expansion of CD14^+^CD11c^+^ macrophages within VA and, to a lesser extent, in noVA tissues, compared to HC (Fig. 3d). These CD11c^+^ macrophages expressed higher levels of CCR2, when compared to their CD11c^−^ counterparts and have been postulated to be newly recruited and pro-inflammatory [38]. Interestingly, by immunofluorescence microscopy, we observed that CD8^+^ T cells and macrophages co-localized within the inflamed regions of the CVID VA duodenum (Fig. 3e).

Next, we searched for altered pathways in CVID enteropathy by Gene Ontology and observed that the transcriptome of both CVID groups was enriched for pathways involved in immune activation and inflammation (Fig. 3f). Differentially induced signatures included several T cell–related pathways, resulting from differential expression of genes such as *EOMES*, *GZMB*, *GZMA*, *TIGIT*, *PDCD1*, and *IL-15*, among others (Fig. S3c). While no significant differences in the inflammatory pathways between VA and noVA tissues was detectable, VA tissues still revealed a more profound upregulation of genes related to the inflammatory response pathway (Fig. 3f). Additionally, both CVID groups presented with an upregulation of *LCN2*, a marker for intestinal inflammation [39], and *HLA-E*, among other MHC molecules (Fig. S3d). Especially the antiviral and IFN type I/III and II response pathways were highly enriched in both CVID groups, when compared to HCs. Downregulated pathways mostly related to digestion, lipid metabolism, and carbohydrate metabolism, indicating an impaired metabolic function of the intestinal epithelium (Fig. 3f and Fig. S3e). Whereas intestinal homeostasis was severely impaired in both CVID groups, integrin, focal adhesion, and WNT signaling were merely downregulated in VA tissues, presumably reflecting the loss of epithelial layer associated with VA (Fig. 3f and Fig. S3f).

### IFN Response Gene Cluster Identifies VA in CVID at Transcription Level and Reveals Already an Induced IFN Response in CVID Patients with Histologically Undetectable Inflammation

**Fig. 4:**
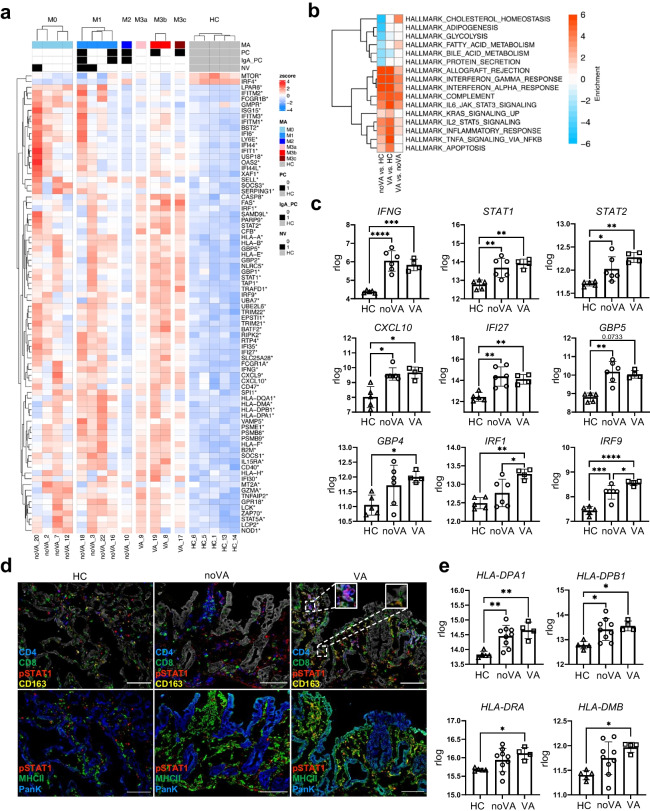
Strong induction of interferon signaling and interferon response genes (IRGs) in CVID VA and norovirus positive tissues. **a** Interferon heatmap showing strong upregulation of interferon signaling in CVID (*n* = 13) compared to HC (*n* = 5) tissues. Genes annotated with interferon type I, II, or III in MSigDB were pooled. Only significantly regulated genes are shown (adjusted *P* value < 0.05). Color code represents the Z-score mRNA intensity. Legend depicts information on the Marsh score (M, M0–M3c), presence of total plasma cells (PC) or IgA^+^ PCs (IgA_PC), and presence of norovirus (NV) infection for each patient. **b** Gene set enrichment heatmap showing the regulated terms in VA (*n* = 4) versus noVA (*n* = 6), excluding NV^+^ samples. Top 10 significant HALLMARK gene sets per comparison are shown. Color code represent the enrichment score (i.e., − log10 *P* value for upregulated gene set, + log10 *P* value for downregulated gene sets). **c** Regularized logarithmic (rlog) counts of *IFNG* and several IRGs from RNA-seq data of HC (*n* = 5), noVA (*n* = 6), and VA (*n* = 4) tissues, excluding NV^+^ samples. **d** Representative image of IF staining performed by MELC for pSTAT1 (in *red*), CD4 (in *blue*), CD8 or HLA-DR (MHCII) (in *green*), and CD163 (in *yellow*) within HC (*n* = 1), noVA (*n* = 1), and VA (*n* = 1) tissues. PanK is shown in *white* in the *upper panel* and in *blue* in the *bottom*. *Dotted line* marks selected fields for higher magnification. *Scale bars* show 100 µm. **e** Regularized logarithmic (rlog) counts of genes involved in the stabilization and formation of MHCII complexes, from RNA-seq samples of HCs (*n* = 5), noVA patients (*n* = 9), and VA patients (*n* = 4). *P* values as determined by one-way ANOVA with Tukey’s multiple comparison test (**c** *IFNG*, *STAT1*, *STAT2*, *IFI27*, *GBP4*, *IRF1*, *IRF9*; **e** *HLA-DPA1*, *HLA-DPB1*, *HLA-DRA*) or Kruskal–Wallis test with Dunn’s multiple comparison test (**c** *CXCL10, GBP5*; **e** *HLA-DMB*) depending if data were normally distributed or not, comparing the mean of each column with the mean of every other column

Given the enriched IFN type I/III and type II signatures in tissues of CVID enteropathy patients, we investigated these pathways more closely. A large number of IFN response genes (IRGs), among the previously identified DEGs, were stratified together with information on NV status, the presence of either total or IgA^+^ PCs, as well as the Marsh-Oberhuber score on single patient-derived tissues (Fig. 4a). This suggested that beside an increase in Marsh-Oberhuber scoring, also NV infection may have a strong influence on the induction of several IRGs within the tissue. Therefore, pathway analysis was performed after exclusion of NV^+^ samples, in order to eliminate the possible impact of norovirus infection on gene set differences associated with the presence or absence of VA. This revealed a greater enrichment of genes assigned to complement, apoptosis, and IFN type I/III and II responses in VA, compared to noVA tissues (Fig. 4b). Tissues of both noVA and VA groups presented with a significantly increased mixed IFN type I/III and II signature, including *IFNG* itself, *STAT1*, *STAT2*, *CXCL10*, Interferon Alpha Inducible Protein* 27* (*IFI27*), *GBP5*, *GBP4*, *IRF1*, and *IRF9* (Fig. 4c). *IRF1* and *IRF9* were the only differentially expressed IRG transcripts differentially expressed in the bulk RNA-seq between VA and noVA tissues. These findings were verified by RT-qPCR in a larger number of patient (*n* = 27–31) and HC (*n* = 10) samples (for further information on included patients and HCs, see Table S1). Within the larger group investigated by RT-qPCR, also *STAT1*, *CXCL10*, *IFI27*, *GBP5*, *IRF1*, *IFI35*, and *ISG15* expression was significantly increased in VA when compared to noVA tissues (Fig. S4a). Although the few patients with defined monogenetic defects did not allow for reliable conclusions, it seemed that CTLA-4 and possibly NFKB1 insufficiency may predispose for an elevated expression of some of the investigated IRGs within small intestinal tissues (Fig. S4a). Furthermore, we asked whether the mixed IFN type I/III and II signature would already be visible in tissues without any histological evidence of inflammation (Marsh-Oberhuber score 0). Indeed, *IFNG*, *STAT2*, *CXCL10*, *IFI27*, *GBP5*, and *IRF9* were significantly upregulated in the patient samples with a Marsh score of 0 compared to HCs (Fig. S4b). Given the increased *STAT1* transcript expression in CVID enteropathy samples, especially in VA tissues, and its important role in IFN signaling, we visualized pSTAT1 by MELC. An increased expression was observed in the VA tissue, locating especially to sub-epithelial CD4^+^ T cells and CD8^+^ T cells that clustered with macrophages, suggesting active STAT1 signaling in these cells (Fig. 4d, *upper row*). Although quantification of relative cell counts of pSTAT1^+^ cells among total, CD4^+^, and CD8^+^ T cells within VA in comparison to HC tissues did not reach significance (Fig. S4c), the analysis of the MFI of pSTAT1 within single-CD8 T cells of VA and noVA tissues showed significantly higher pSTAT1 expression levels in VA and noVA tissues compared to HC-derived tissues (Fig. S4d).

In addition, as IFN-γ has previously been attributed to induce major histocompatibility complex class II (MHCII) expression on small intestinal epithelial cells [40] and active CD tissues present with increased MHCII expression in the villi area [41], its expression and localization was investigated. Strikingly, in the VA tissue, enterocytes of the villi area highly expressed MHCII, which even extended to the crypts, whereas in the noVA tissue, expression was weaker and mainly detected on the villi (Fig. 4d, *lower row*). This finding was further supported by a highly induced expression of several genes, which are involved in the expression and stabilization of MHCII molecules within VA and for *HLA-DPA1* and *HLA-DPB1* also within noVA, compared to HC tissues (Fig. 4e). Moreover, analysis of pSTAT1 protein levels within single intestinal epithelial cells (IECs) by MELC showed highly induced pSTAT1 expression within IECs of VA, compared to noVA and HC tissues (Fig. S4e), although not reaching expression levels of intestinal T cells (data not shown).

### *The Lack of IgA*^+^*and Total Plasma Cells in CVID Enteropathy Is Associated with Increased Inflammation and Loss of Tissue Homeostasis*

**Fig. 5:**
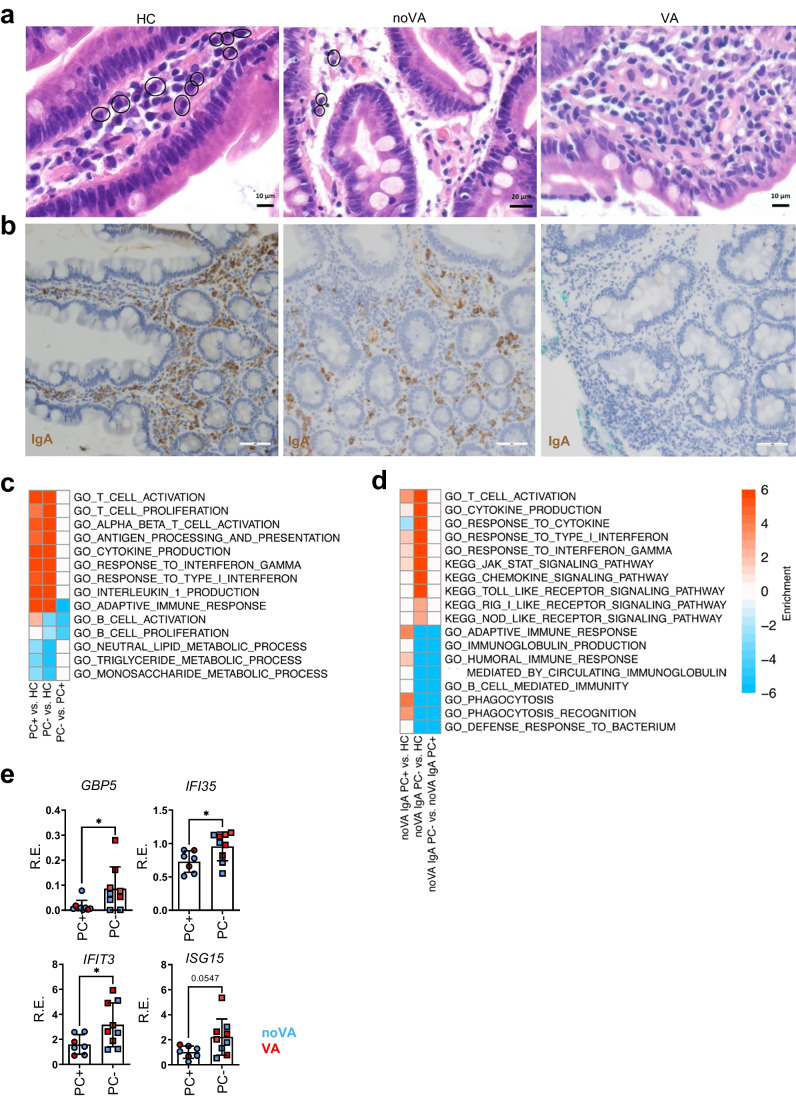
The lack of total and IgA^+^ plasma cells (PCs) contributes to tissue inflammation and impaired metabolic function in CVID enteropathy patients. **a** Representative histologies for the presence of PCs in duodenal biopsies of HC, a noVA patient, and a VA patient. *Circles* denote plasma cells. **b** Representative histologies for the presence of IgA^+^ PCs in duodenal biopsies of a HC, a noVA patient, and a VA patient, performed by IHC. *Scale bars* show 100 µm. **c** Gene set enrichment heatmap showing the differentially regulated terms in PC^+^ (*n* = 5) to PC^−^ (*n* = 8) tissues. Relevant gene sets were selected among the top significant ones. Color code represents the enrichment score. **d** Gene set enrichment heatmap showing the differentially regulated terms in noVA IgA PC^+^ (*n* = 3) to noVA IgA PC^−^ (*n* = 6) tissues. Relevant gene sets were selected among the top significant ones. Color code represents the enrichment score. **e** Comparison of the RE of several IRGs in PC^+^ (*n* = 7) to PC^−^ (*n* = 9) tissues, analyzed by RT-qPCR, excluding NV^+^ tissues and tissues from patients with monogenetic defects. Color code indicates noVA (in *blue*) and VA (in *red*) tissues. *P* values as determined by unpaired *t* test (**e** *GBP5*, *IFI35*, *IFIT3*, *ISG15*) as data was normally distributed

As the absence of IgA contributes to an altered mucosal homeostasis and induced inflammation [16], we investigated the impact of the absence of total and IgA^+^ PCs on the tissue transcriptome. PCs of all subclasses were either reduced or completely absent in the majority of VA and noVA tissues (Fig. 5a and Fig. 1d). Whereas some noVA patients still presented with intestinal IgA^+^ PC, all VA patients lacked this subset (Fig. 5b and Fig. S5a). Pathway analysis, based on the presence or absence of total PCs, displayed a similar induction of T-cell activation and mixed IFN type I/III and II responses, with concurrent downregulation of metabolic pathways between both groups, whereas metabolic pathways were more severely downregulated in PC^−^ tissues (*n* = 8), then PC^+^ (*n* = 5) tissues when compared to HCs (*n* = 5), respectively (Fig. 5c). As for IgA^+^ PCs, the comparison of noVA IgA^−^ PC (*n* = 6) to noVA IgA^+^ PC (*n* = 3) tissues showed no exclusively dysregulated pathway, besides the expected downregulation of B-cell and immunoglobulin-related pathways in tissues lacking IgA^+^ PCs. However, the comparison of noVA tissues that lacked IgA^+^ PCs to HC tissues displayed a stronger upregulation of genes involved in T-cell activation and cytokine production, as well as mixed IFN type I/III and II responses, than the comparison of noVA tissues containing IgA^+^ PC to HC tissues (Fig. 5d). Interestingly, in comparison to HCs, solely noVA tissues lacking IgA^+^ PCs presented with an upregulation of Toll-like, RIG-I-like, and NOD-like receptor signaling pathways, indicating a stronger activation of innate mucosal responses (Fig. 5d). In order to investigate the impact of the absence of tissue homing PCs on the observed IFN-driven inflammation, we assessed selected IRGs by RT-qPCR in a higher number of samples (PC^+^, *n* = 7; PC^−^, *n* = 9; information on patients is listed in Table S1). This revealed a significantly higher expression of *IFI35*, *GBP5*, and *IFIT3* in tissues lacking total PCs (Fig. 5e), whereas no significant differences were detectable comparing tissues with and without IgA^+^ PCs directly (Fig. S5b).

### NV Infection Augments the IFN Signature in CVID Enteropathy Patients

**Fig. 6:**
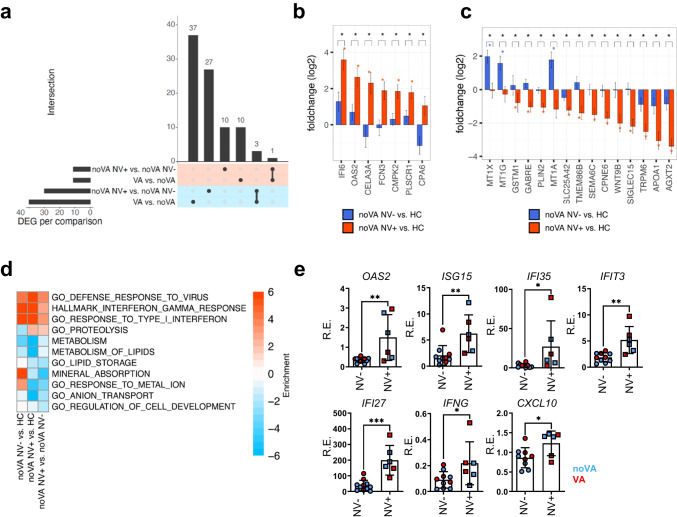
NV infection exacerbates the mixed IFN type I/III and II signature in CVID enteropathy and contributes to the impairment of homeostatic tissue functions. **a** UpSet plot comparing the differentially expressed genes (adjusted *P* value < 0.05) in noVA NV^+^ (*n* = 3) vs. noVA NV^−^ (*n* = 3) tissues and in VA (*n* = 4) vs. noVA (*n* = 6) tissues. Upregulated and downregulated genes are highlighted with *red* and *blue background*, respectively. **b** Gene-level log2 fold change bar plots for differentially upregulated genes in noVA NV^+^ (*n* = 3, in *red*) vs. noVA NV^−^ (*n* = 6, in *blue*) samples. **c** Gene-level log2 fold change bar plots for differentially downregulated genes in noVA NV^+^ (*n* = 3, in *red*) vs. noVA NV^−^ (*n* = 6, in *blue*) samples. **d** Gene set enrichment heatmap showing the regulated terms in noVA NV^+^ (*n* = 3) vs. noVA NV^−^ (*n* = 6) tissues. Relevant gene sets were selected among the top significant ones. Color code represent the enrichment score (i.e., − log10 *P* value for upregulated gene set, + log10 *P* value for downregulated gene sets). **e** Relative expression (RE) of the antiviral response genes *OAS2*, *ISG15*, *IFI35*, and *IFIT3* among other IRGs, analyzed by RT-qPCR. Expression was analyzed within NV^+^ (*n* = 6) and NV^−^ (*n* = 9–11) tissues, excluding tissues of patients with monogenetic defects. Color code indicates noVA (in *blue*) and VA (in *red*) tissues. *P* values as determined by unpaired *t* test (**e** *OAS2*, *IFIT3*, *IFI27*) or Mann–Whitney test (**e** *ISG15*, *IFI35*, *IFNG*, *CXCL10*) depending if data were normally distributed or not

Since NV was previously postulated to drive VA in CVID and is capable of stimulating IFN responses in host intestine [42, 43], we investigated the effect of NV infection on the duodenal transcriptome of our patients. As our RNA-seq cohort contained NV^+^ patients only among the less severe disease (noVA) group, we compared noVA NV^+^ (*n* = 3) to noVA NV^−^ (*n* = 6) tissues. Ten genes appeared to be specifically upregulated within NV^+^ tissues (Fig. 6a), including the IRGs *IFI6*, *OAS2*, *CMPK2*, and *PLSCR1* (Fig. 6b). Additionally, NV^+^ tissues showed 27 downregulated DEGs, with many of them being involved in homeostatic and ion metabolic functions (Fig. 6c). Pathway analysis revealed a stronger expression of genes involved in defense response to virus and IFN type I/III and II response pathways in the NV^+^ compared to NV^−^ samples. Downregulated pathways included mainly metabolic functions, like lipid metabolism and storage, as well as homeostatic functions, like ion homeostasis or cell development (Fig. 6d). In order to confirm the altered mixed IFN type I/III and II signature upon NV infection, we performed RT-qPCRs for selected IRGs in a higher number of samples (NV^+^, *n* = 6; NV^−^, *n* = 9–11; further information on patients is listed in Table S1). Here, the antiviral type I IRGs *OAS2*, *ISG15*, *IFI35*, and *IFIT3* were significantly increased in NV^+^ compared to NV^−^ tissues (Fig. 6e), more frequently resulting from differences in the expression between NV-positive and NV-negative noVA tissues (Fig. S6a). Interestingly, the upregulation of *IFI27* and *CXCL10* expression, which we had previously detected especially in tissues with VA, was also induced in noVA tissues by chronic NV infection (Fig. S6a), suggesting an exacerbated pathological mixed IFN type I/III and II signature as a potential driver of norovirus-positive CVID enteropathy.

## Discussion

Enteropathy in CVID patients is frequently associated with malabsorption, inflammation, and immune dysregulation and substantially contributes to higher morbidity in the affected patients. Whereas duodenal pathology is reminiscent of celiac manifestations in immunocompetent hosts, the different pathogenic factors in CVID enteropathy are still not well defined.

In this study, we demonstrate that the expansion of cytotoxic active CD8 T cells and the prominent mixed IFN type I/III and II signature in CVID enteropathy is associated with the different histological stages of the disease and it can be exacerbated by concomitant NV infection especially during the noVA stage. Extending previous reports [44], duodenal tissues of our CVID enteropathy patients displayed increased T-cell infiltration and a disturbed T-cell homeostasis, characterized by a significant decrease in TH17 and an expansion of TH1 cells, corroborated by a strong induction of IFN-γ on transcript and protein level within a total tissue of CVID VA patients. In celiac disease (CD), gliadin-specific cells have been identified as IFN-γ-producing TH1 cells [45, 46], but inflammatory bystander CD4^+^ T cells have been reported to secrete high amounts of IL-17 during active inflammation [45] and at more severe disease stages, due to the loss of villous architecture and intestinal barrier integrity, allowing for increased bacterial translocation [47–49]. In CVID, the lack of IL-17-producing cells may origin from a locally driven shift from bacterial-induced TH17 cells to double-producing IFN-γ/IL-17 and finally IFN-γ single-producing TH1 cells [50], which by itself may contribute to the failure of local IgA production [50–54] and together to the observed intestinal pathology [55].

Differentially regulated pathways in noVA and VA tissues simultaneously highlighted signs of T-cell activation and exhaustion, recently also debated in circulating T cells of CVID patients with immune dysregulation [56]. Compatible with the authors’ conclusion, we show elevated Eomes and GzmB expression in tissue-resident memory-like CD8^+^CD103^+^ IELs, which is more compatible with an active cytotoxic, than exhausted behavior in the ongoing tissue destruction of CVID VA [57, 58]. Consistently, *GZMB* was a major dysregulated gene, differentiating VA as well as noVA from HC samples, and pathway analysis indicated “Granzyme mediated apoptosis” as a major upregulated pathway in noVA samples. At the transcriptional level, expression discriminated already tissues of patients without visual inflammation (Marsh 0) from HC tissues, suggesting *GZMB* as a potential biomarker for active enteropathy without histological signs of inflammation. However, looking at GzmB protein expression, exclusively VA tissues presented with highly increased numbers of GzmB^+^CD103^+^CD8^+^ T cells. NV infection presented with high percentages of total, CD8^+^, and CD4^+^TH1 cells as well as exacerbated *GZMB* transcript and protein expression, indicating the expansion of a rather activated than exhausted T-cell phenotype upon NV infection in the affected tissues [37]. The observed difference in mRNA versus protein expression likely depicts that armed cytotoxic CD8^+^ T cells are mainly present within VA- or NV-infected CVID enteropathy tissues, whereas resting cytotoxic T cells seem already present within histologically less inflamed and NV-negative tissues [59].

The increased cytotoxicity was further associated with an enhanced transcriptional profile of pro-inflammatory M1 macrophages in VA and, to a lesser extent, in noVA tissues, as suggested previously [16]. Macrophages represent essential gate keepers of the mucosal homeostasis and intestinal epithelial barrier integrity [60], partly by building the first line of defense against invading pathogens [61]. Their high plasticity, either executing non-inflammatory clearance of bacteria and debris [62] or aggravating inflammation as TLR-responsive pro-inflammatory cells [63], contributes to both tissue homeostasis and destruction. The gradual increase of CD14^+^CD11c^+^CCR2^high^ pro-inflammatory macrophages from noVA to VA tissues, as the main TLR-responsive and cytokine-producing subset in steady-state duodenal tissue [64], has also been observed to accumulate under inflammatory conditions as IBD [38] and CD [65, 66] or during colitis [67], potentially recruited via CCR2 expression [61]. These cells most likely contribute to the observed IFN-driven inflammation and local accumulation of CD8^+^ IELs at the site of tissue destruction through their capacity to produce *CXCL9* and *CXCL10* [68–73]. Together, this data suggests an important role of the interaction between newly recruited pro-inflammatory macrophages and cytotoxic CD8 T cells in the aggravation of enteropathy in CVID.

All these changes take place in an environment of strongly induced mixed IFN type I/III and II signatures, along with a severe impairment of metabolic and homeostatic functions. The coherence of enteropathy, inflammatory immune alterations, and impairment of metabolic functions has been reported in CVID patients and mice [16, 17], but not investigated in relation to the potential contributing factors or the additional impact NV infection might have.

The mixed IFN type I/III and II signature in CVID enteropathy does not require VA or NV infection as certain IRGs were already induced in noVA tissues or even in tissues without histological signs of inflammation (Marsh 0). Similar to celiac disease [74], expression levels of *GBP5*, *CXCL10*, *IFI27*, and *IFNG* were increased in tissues of patients with no or low-grade intestinal injury. The increased IFN-driven transcriptional profile was further associated with high STAT1 protein phosphorylation in CD8^+^ T cells of VA tissues, indicating active IFN signaling especially in lamina propria homing CD8^+^ T cell-macrophage clusters, supporting the idea of an active IFN-γ-driven cross talk of these cells in CVID VA tissues. Compatible with this interpretation was the high expression of *GBP5* and *IRF1* in VA, which was not further induced by NV, since *GBP5* is induced in the M1 subset upon LPS/IFN-γ stimulation, upon HIV infection [75, 76] and in active Crohn’s disease and ulcerative colitis (UC) patients [77]. *IRF1* is reported also to be highly expressed in celiac disease and being important for robust TH1-macrophage responses [78, 79]. Upregulation of STAT1 phosphorylation was also seen in intestinal epithelial cells of VA tissues, which was associated with an increased MHCII expression of epithelial cells, reflecting an IFN-γ-driven inflammation of the GI barrier.

The absence of mucosal IgA^+^ or total PCs was previously linked to an increased mixed IFN type I/III and II signature in CVID-derived duodenal tissues [16, 17]. Our histological phenotyping confirmed a correlation between the presence of VA and the lack of total, especially IgA^+^ PCs. When comparing transcriptional signatures to HC, T-cell activation, pro-inflammatory cytokine, chemokine and JAK/STAT signaling, as well as IFN responses were more strongly upregulated in the absence of IgA^+^ PCs, compared to tissues with IgA^+^ PCs, although the direct comparison between both cohorts did not reach significance. Furthermore, TLR, NOD-like, and RIG-I-like receptor signaling pathways were upregulated only in tissues lacking IgA, suggesting an excessive innate immune response due to an unrestrained exposure of epithelial and immune cells to commensal antigens, tipping the balance of the mucosal immune system towards a pro-inflammatory state [80].

While our study was not aiming to identify risk factors for chronic NV infection in CVID, it is remarkable that the prevalence of chronic NV infection seems higher in patients lacking PCs in the gastrointestinal mucosa as 12 out of 13 NV-positive patients (92%) in contrast to 24/29 NV-negative patients (83%) lacked mucosal IgA PCs and 10/13 NV-positive patients (76%) vs. 14/29 NV-negative patients (48%) all mucosal PCs (data not shown, for further information, see Table S1).

The investigation of the impact of chronic NV infection on the local IFN signature revealed a strong overlap of signatures, but also some detectable differences. Thus, NV infection was not only associated with, but seemed able to enhance type I/III and II IFN responses, including transcripts of *IFI27* and *CXCL10*, and aggravated metabolic dysfunction when compared to disease stage–matched NV^−^ samples in our cross-sectional analysis. A potential source of the elevated IFN type II signature in NV^+^ patients represents the local expansion of effector T cells, resembling T_RM_ (CD103^+^CD8^+^GzmB^+^) T cells, producing high amounts of IFN-γ and TNF-α [81]. Increased transcripts of *IFI27* within VA and slightly less in noVA tissues, also reported in rheumatoid arthritis (RA), psoriasis [82, 83], and UC [84], were especially observed in NV^+^ tissue samples and probably reflect a mixed response of IFN type I/III in an IFN-γ/TNF-α-enriched environment [83]. Compatible with a prominent induction of IFN type I responses by NV infection, the IRGs *OAS2*, *ISG15*, and *IFIT3* were significantly increased in NV-positive noVA tissues. This finding reflects observations that were previously made by infecting intestinal organoid cultures with human norovirus strains [85]. A limitation of our study is that these findings were not made longitudinally in single patients before and after NV infection.

In summary, CVID patients with duodenal enteropathy present with a locally altered T-cell homeostasis and increased cytotoxicity, associated with a TH1 and inflammatory macrophage–driven mixed IFN type I/III and II signature. These signatures are already present at mild stages of the disease, and their extent reflects the progression of the histopathological changes. This association is affected by contributing factors, like the absence of IgA PCs and possibly certain genetic backgrounds, and it is especially aggravated by concomitant NV infection.

Our findings therefore support the need for novel antiviral drugs to eliminate chronic norovirus infection, and meanwhile T-cell targeting therapies like calcineurin inhibitors or potentially the use of JAK inhibitors, in order to control the local inflammation.

## Supplementary Information

Below is the link to the electronic supplementary material.

Supplementary file1 (DOCX 2.32 MB)

## Data Availability

The RNA-seq data that support the findings of this study are available in the GEO repository under accession number GSE189820. Token: odsdewconxqrhuz. Further data and material data are available from the authors upon specific request.
