# Supplementary material for: Interferon-Driven Immune Dysregulation in Common Variable Immunodeficiency–Associated Villous Atrophy and Norovirus Infection
Source: J Clin Immunol. 2022 Oct 25;43(2):371–90. doi: 10.1007/s10875-022-01379-2 (PMC9892141; doi:10.1007/s10875-022-01379-2)
Supplement: Supplementary file 1 — Supplementary file1 (DOCX 2.32 MB) [file 10875_2022_1379_MOESM1_ESM.docx]

Supplementary Tables

**Table S1.** Key clinical and histological characteristics of the cohort.

HC, healthy controls; age is indicated in years; f, female; m, male; VA, villous atrophy; noVA, no villous atrophy; M, Marsh-Oberhuber Score; NV, norovirus; HP, Helicobacter pylori; o. Inf., other infection; AIC, autoimmune cytopenias; LD, liver disease; RTX, Rituximab in history; s.S., systemic Steroids, l.S., local Steroids; PC, plasma cells (mucosal); IgA/IgG/IgM PCs, IgA^+^/IgG^+^/IgM^+^ plasma cells (in tissue); 1, yes; 0, no, NA, data not available. RNA-seq, RNA sequencing data; qPCR, RT-qPCR data; MELC, Multi-Epitope-Ligand-Cartography data; Fig. xy, data of patients used in the listed figures; x, included; x (+3y), new sample of listed patients was used 3 years after first biopsy.

**Table S2:** Question and diagnosis for upper endoscopic procedure in healthy controls.

HC, healthy control; H.p., Helicobacter pylori;

**Table S3**: List of antibodies used in flow-cytometry assays.

| **Antigen** | **Fluorochrome** | **Clone** | **Company** | **Catalogue Number** |
| --- | --- | --- | --- | --- |
| CCR2 | BV421 | K036C2 | BioLegend | 357210 |
| CCR6 | BV605 | G034E3 | BioLegend | 353420 |
| CD27 | BV605 | L128 | BD | 15832119 |
| CD27 | BV421 | M-T271 | BioLegend | 356418 |
| CD3 | PE-Cy7 | UCHT1 | Beckman Coulter | 6607100 |
| CD3 | PerCP-Cy5.5 | SK7 | BioLegend | 344808 |
| CD3 | AF700 | UCHT1 | BioLegend | 300424 |
| CD4 | PE-Cy7 | RPA-T4 | BioLegend | 300512 |
| CD4 | BV421 | RPA-T4 | BioLegend | 300532 |
| CD8 | AF647 | SK1 | BioLegend | 344726 |
| CD8 | PerCP-Cy5.5 | SK1 | BioLegend | 344710 |
| CD8 | APC-Cy7 | SK1 | BioLegend | 344714 |
| CD11c | AF700 | Bu15 | BioLegend | 337220 |
| CD14 | PE-Cy7 | M5E2 | BD | 557742 |
| CD45 | Pacific Blue | HI30 | BioLegend | 304029 |
| CD45 | BV785 | HI30 | BioLegend | 304048 |
| CD45RA | FITC | ALB11 | Beckman Coulter | A07786 |
| CD45RA | APC-Cy7 | HI100 | BioLegend | 304128 |
| CD45RA | BV605 | HI100 | BioLegend | 304134 |
| CD69 | FITC | FN50 | BD | 555530 |
| CD103 (ITGAE) | PerCP-Cy5.5 | Ber-ACT8 | BioLegend | 350226 |
| CXCR3 | PE | 49801 | R&D Systems | FAB160P |
| CXCR5 | AF488 | RF8B2 | BD | 740266 |
| Granzyme-B | PE | QA16A02 | BioLegend | 372208 |
| HLA-DR | PerCP-Cy5.5 | L243 | BioLegend | 307630 |
| DAPI |  |  | Sigma Aldrich | D8417 |
| IFN-γ | FITC | B27 | BD | 15801569 |
| IL-17A | PE | eBio64DEC17 | eBioscience | 12717942 |
| Zombie Violet |  |  | BioLegend | 423114 |
| Zombie UV |  |  | Biolegend | 423107 |

| **Antigen** | **Fluorochrome** | **Clone** | **Company** | **Catalogue Number** | **RRID** |
| --- | --- | --- | --- | --- | --- |
| CD3 | PE | REA613 | Miltenyi Biotec | 130-113-139 | AB_2725967 |
| CD4 | PE | VIT4 | Miltenyi Biotec | 130-113-776 | AB_2726025 |
| CD8 | PE | BW135/80 | Miltenyi Biotec | 130-113-720 | AB_2726261 |
| CD45 | PE | 5B1 | Miltenyi Biotec | 130-113-118 | AB_2725946 |
| CD103 | PE | Ber-ACT 8 | Miltenyi Biotec | 130-103-709 | AB_2654387 |
| CD163 | PE | RM3/1 | BioLegend | 333605 | AB_1134005 |
| Eomes | PE | WD1928 | BD | 12-4877-41 | AB_2572881 |
| Granzyme-B | PE | REA226 | Miltenyi Biotec | 130-116-654 | AB_2727639 |
| HLA-DR,-DP,-DQ | PE | REA332 | Miltenyi Biotec | 130-120-789 | AB_2752176 |
| Pancytokeratin | PE | AE1 + AE3 | Arigo Biolaboratories | ARG56130 | NA |
| pSTAT1 | PE | REA159 | Miltenyi Biotec | 130-105-471 | AB_2653564 |
| VCAM | PE | REA269 | Miltenyi Biotec | 130-121-992 | AB_2801820 |
| DAPI |  |  | Roche | 10236276001 | NA |

**Table S4**: List of antibodies used in MELC staining assays.

| **Gene** | **Forward Primer Sequence** | **Forward Primer Sequence** |
| --- | --- | --- |
| CXCL10 | GGTGAGAAGAGATGTCTGAATCC | GTCCATCCTTGGAAGCACTGCA |
| GBP5 | GGAGCCTCGGAAAGGAATAC | GCTCGTTCTGCCTTTGAATC |
| GZMB | GGAGGCCCTCTTGTGTGTAA | ATTACAGCGGGGGCTTAGTT |
| IFI35 | CCCACAGCCTCATCTTGAGT | TCTGAAGCCTCAGCTCTTGC |
| IFIT3 | TGAGGAAGGGTGGACACAAC | ACATCGCAATTGCCAGTCCA |
| IFI27 | ATCAGCAGTGACCAGTGTGG | TGGCCACAACTCCTCCAATC |
| IFNG | CTAGGGATGGAGTGGCTCAG | TCAGGAATGTTGAGCACCAG |
| IRF1 | AGCTCAGCTGTGCGAGTGTA | TAGCTGCTGTGGTCATCAGG |
| ISG15 | GAGAGGCAGCGAACTCATCT | CTTCAGCTCTGACACCGACA |
| OAS2 | ACAGCTGAAAGCCTTTTGGA | GCATTAAAGGCAGGAAGCAC |
| STAT1 | CCGTTTTCATGACCTCCTGT | TGAATATTCCCCGACTGAGC |
| STAT2 | GAGGCCTCAACTCAGACCAG | GCGTCCATCATTCCAGAGAT |
| TUBA1 | GACCAAGCGTACCATCCAGT | CACGTTTGGCATACATCAGG |
| UBC | CCACTCTGCACTTGGTCCTG | TGCAACAACTTTATTGAAAGGAAA |

**Table S5**: Primer for RT-qPCR experiments.

****Supplementary Figures

**Figure S1. Multi-dimensional analysis of CVID noVA and VA patients by clinical, histological and flow-cytometry and transcriptome data.**

a) Frequency of clinical manifestations among noVA (n=51) and VA (n=21) patients. *Black bars* represent percentage of patients, being positive for listed criteria.

b) Frequency of histological manifestations among the initial cohort of noVA (n=17) and VA (n=13) patients, investigated by IHC. Black bars represent percentage.

c) Gating strategy for T cell phenotyping, as determined by CD4 and CD8 expression. Pre-gated on living CD45^+^ lymphocytes.

d) Proportion of IFN-γ^+^/IL17A^+^ and IFN-γ^+^/IL17A^-^ cells of memory CD4^+^ T cells, after 4hrs PMA + Ionomycin stimulation, isolated from tissues of HCs (n=8), noVA (n=10) and VA (n=11) patients. Patients with Norovirus infection are highlighted *in red.*

e) Gating strategy for cytokine production of memory CD4^+^ T cells, after 4hrs PMA+Ionomycin stimulation. Pre-gated on living lymphocytes by FSC/SSC.

f) Proportion of TH17, TH1, TH2 and TH1/17 subsets of memory CD4^+^ T cells within duodenal tissues of HCs (n=10), noVA (n=10) and VA (n=12) patients. Patients with Norovirus infection are highlighted *in red*.

g) Gating strategy for TH cell subsets, as defined by CXCR3 and CCR6 expression and pre-gated on living CD45^+^ lymphocytes.

P values as determined by one-way ANOVA with Tukey´s multiple comparison test (d, f, TH1, TH17, TH1/17) or Kruskal-Wallis test with Dunn’s multiple comparisons test (f, TH2), depending if data were normally distributed or not, comparing the mean of each column with the mean of every other column.

**a**

**b**

*GZMB* (R.E.)

VA

noVA

HC

**NV+**

**NFkB1/NV+**

**NFkB1**

**CTLA4**

**PI3K**

**FAS**

**ICOS**

**TACI**

*GZMB* (R.E.)

M0

HC

**Figure S2. Increased transcript expression of Granyzme-B in CVID-derived noVA and VA tissues.**

a) Relative expression (R.E.) of *GZMB* analyzed by RT-qPCR within tissues of HCs (n=10), noVA (n=22) and VA (n=16) patients. Color code indicates tissues of patients with NV infection (*in red*) and several monogenetic defects.

b) Statistical analysis comparing R.E. of *GzmB* within tissues of HCs (n=10) to CVID noVA patients without detectable T-cell infiltration (Marsh 0, n=8).

P values as determined by Kruskal-Wallis test with Dunn’s multiple comparisons test (a), comparing the mean of each column with the mean of every other column. Mann-Whitney test was used to define P value of unpaired two-column comparison (b).

**b**

**a**


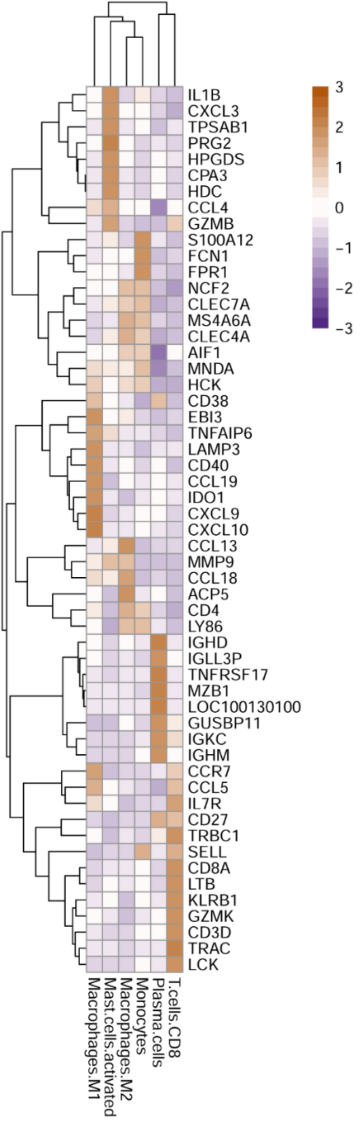

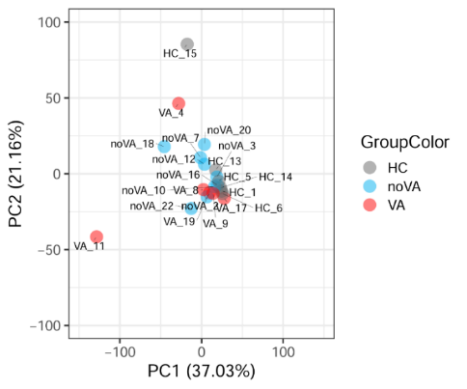


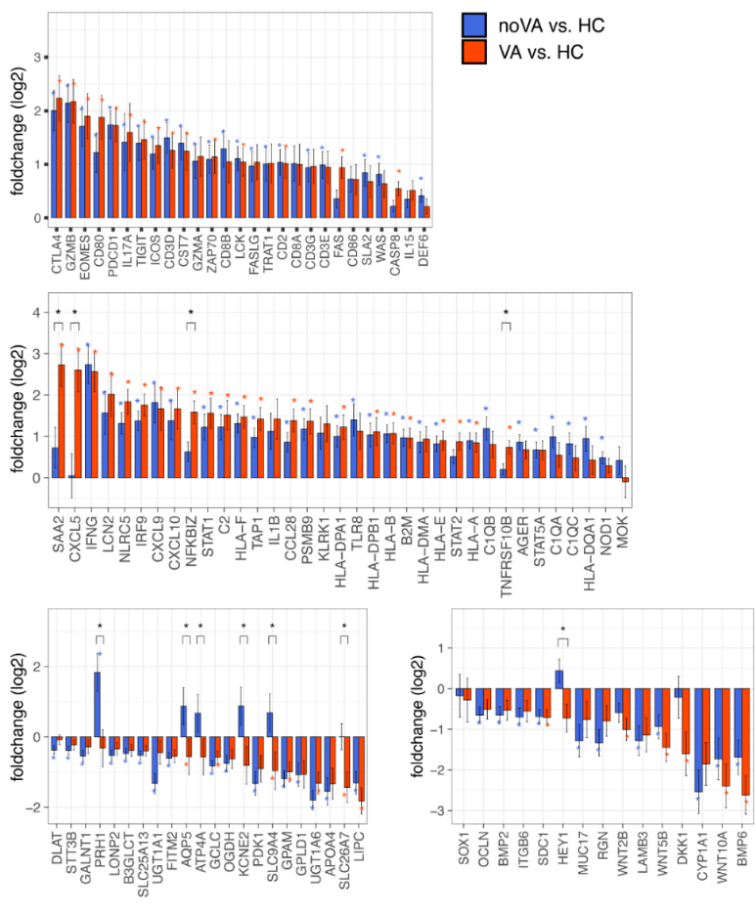


**c**

**d**

**f**

**e**

**Figure S3. Increased inflammation and immune activation at transcriptional level in VA and noVA tissues of CVID patients**

a) Principal Component Analysis (PCA) on RNA-seq complete dataset. Three outlier samples (HC_15, VA_4 and VA_11) were identified and discarded from further analysis.

b) Cell type-specific signature heat map from the immune cell deconvolution. Color code indicates the column-wise scaling of gene weight for each cell type.

c-f) Gene-level log2 fold change bar plots (c, T cells; d, Immune activation and inflammation; e, Digestion; f, Homeostasis). Genes are sorted according to their log2 fold change in VA vs. HC.

Significant differences in the altered regulation between noVA vs. HC and VA vs. HC are labeled with an asterisk (“*”).

*STAT1* (rlog)

HC

M0

*IFNG* (rlog)

HC

M0

*IFNG* (R.E)

*STAT1* (R.E)

*CXCL10* (R.E)

HC

noVA

VA

HC

noVA

VA

HC

noVA

VA

**b**

**a**

*STAT2* (rlog)

HC

M0

HC

noVA

VA

HC

noVA

VA

HC

noVA

VA

*IFI27* (R.E)

*IRF1* (R.E)

*GBP5* (R.E)

HC

M0

*GBP5* (rlog)

*IFI27* (rlog)

HC

M0

*CXCL10* (rlog)

HC

M0

*IFI35* (R.E)

NFkB1

CTLA4

PI3K

FAS

ICOS

TACI

*ISG15* (R.E)

HC

noVA

VA

HC

noVA

VA

*IRF9* (rlog)

HC

M0

*IRF1* (rlog)

HC

M0

*GBP4* (rlog)

HC

M0

counts/100 IECs

pSTAT1^+^CD8^+^ T cells

HC

noVA

VA

counts/100 IECs

pSTAT1^+^CD4^+^ T cells

HC

noVA

VA

**e**

**d**

**c**

********

HC

noVA

VA

pSTAT1^+^ IECs

MFI per cell

counts/100 IECs

pSTAT1^+^ T cells

HC

noVA

VA

pSTAT1^+^ CD8^+^ T cells

MFI per cell

HC

noVA

VA

**Figure S4. Strong induction of Interferon Response Genes (IRGs) in VA tissues and already upregulated IFN responses in lower Marsh score tissues.**

Relative expression (R.E.) of *IFNG* and several IRGs, analyzed by RT-qPCR, within HCs (n=10), noVA (n=18-21) and VA (n=9-10) tissues within the expanded cohort, excluding NV^+^ samples. Color code indicates tissues derived from patients with various monogenetic defects.

b) Regularized logarithmic (Rlog) counts of *IFNG* and several IRGs genes from RNA-seq data, comparing HC tissues (n=5) to noVA tissues with histologically undetectable inflammation (Marsh 0, n=3), excluding NV^+^ samples.

c) Relative counts of pSTAT1^+^ total T cells, pSTAT1^+^CD4^+^ T cells and pSTAT1^+^CD8^+^ T cells per 100 intestinal epithelial cells (IECs) within HCs (n=3), noVA (n=3) and NV^+^ VA (n=3) tissues. Two field of view (FOV) are shown for each patient (except 1 noVA and 1 VA tissue). Color code indicates corresponding FOVs (white, tissue 1; grey, tissue 2; black, tissue 3) acquired from the same sample. *In red* NV positive patients.

d) Median fluorescent intensity (MFI) of pSTAT1 within pSTAT1^+^CD8^+^ cells of HCs (n=3, total cell count=1900), noVA (n=3, total cell count=2502) and VA (n=3, total cell count= 2453) tissues. *In red* NV positive patients.

e) Median fluorescent intensity (MFI) of pSTAT1 within pSTAT1^+^ intestinal epithelial cells (IECs) of HCs (n=3, total cell count= 1817), noVA (n=3, total cell count= 1909) and VA (n=3, total cell count= 2615) tissues. *In red* NV positive patients.

P values as determined by one-way ANOVA with Tukey´s multiple comparison test (a, *IFI35*; c, CD4^+^ T cells, CD8^+^ T cells) or Kruskal-Wallis test with Dunn’s multiple comparisons test (a, *IFNG, STAT1, CXCL10, IFI27, GBP5, IRF1, ISG15;* c, total T cells; d; e) depending if data were normally distributed or not, comparing the mean of each column with the mean of every other column. For unpaired two grouped comparisons P values as determined by unpaired t test (b, *IFNG,* *STAT1*, *STAT2, CXCL10, IFI27, IRF1*) or Mann-Whitney test (b, *GBP5, IRF9*) depending if data were normally distributed or not.


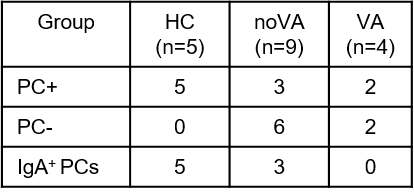


**a**

**b**

*CXCL10* (R.E.)

*IFNG* (R.E.)

*ISG15* (R.E.)

noVA

VA

IgA+ PC

IgA- PC

IgA+ PC

IgA- PC

IgA+ PC

IgA- PC

**Figure S5. The lack of total and IgA^+^ plasma cells (PCs) contributes to IFN driven inflammation in CVID enteropathy patients.**

a) Presence of total or IgA^+^ PCs within tissues of the RNA-seq cohort.

b) Comparison of the R.E. of several IRGs in IgA^+^ PC (n=4) to IgA^-^ PC (n=13) tissues, analyzed by RT-qPCR within the expanded cohort, excluding NV^+^ tissues and tissues from patients with monogenetic defects. Color code indicates noVA (*in blue*) and VA (*in red*) tissues. Only IRGs with almost significant difference between IgA^-^ and IgA^+^ PC tissues are shown.

P values as determined by unpaired t test (b, *CXCL10*, *ISG15*) or Mann–Whitney test (b, *IFNG*), depending if data were normally distributed or not.

NV+

NV-

*OAS2* (R.E.)

NV-

NV+

*OAS2* (R.E.)

NV+

NV-

*ISG15* (R.E.)

NV+

NV-

*ISG15* (R.E.)

NV+

*IFI35* (R.E.)

NV-

NV-

NV+

*IFI35* (R.E.)

**a**

NV-

NV+

*IFIT3* (R.E.)

NV-

NV+

*IFIT3* (R.E.)

NV-

NV+

*IFI27* (R.E.)

NV-

NV+

*IFI27* (R.E.)

NV-

NV+

*IFNG (*R.E.)

NV-

NV+

*IFNG* (R.E.)

NV-

NV+

*CXCL10* (R.E.)

NV-

NV+

*CXCL10* (R.E.)

**Figure S6.** **Norovirus infection induces IFN Type I/III and II response genes mainly in CVID enteropathy patients without VA.**

a) Relative expression (R.E.) of several IRGs within CVID noVA (blue) and CVID VA (red) tissues, comparing patients without norovirus infection (NV^-^, noVA, n=5; VA, n=4) to patients with norovirus infection (NV^+^, noVA, n=3; VA, n=4)

P values as determined by unpaired t test (a, *OAS2*, *IFI35*, *IFIT3*, *IFI27* noVA) or Mann–Whitney test (a, *IFI27* VA, *ISG15*, *IFNG*, *CXCL10*), depending if data were normally distributed or not.
